# Contested and nervous spaces: exploring the environment of healthcare provision for international migrants in the Gauteng province of South Africa

**DOI:** 10.1080/16549716.2024.2422192

**Published:** 2024-11-05

**Authors:** Janine A. White, Laetitia C. Rispel

**Affiliations:** a School of Public Health, Faculty of Health Sciences, University of the Witwatersrand, Johannesburg, South Africa; b Centre for Health Policy & South African Research Chairs Initiative, School of Public Health, Faculty of Health Sciences, University of the Witwatersrand, Johannesburg, South Africa

**Keywords:** Health system responsiveness, social exclusion, migrants, health workers, South Africa

## Abstract

**Background:**

Notwithstanding the global goal of inclusive universal health coverage, and the notion of migrant-sensitive health systems, limited healthcare access or the exclusion of migrants from national health systems persists. South Africa has a rights-based constitution, but there is an inability or a failure of the health system to recognise and address the health needs of migrants.

**Objective:**

To explore the intersection of the environment of healthcare provision for migrants and the everyday practices and behaviours of health workers and patients in the Gauteng province of South Africa.

**Methods:**

The conceptual frameworks of health system responsiveness and social exclusion informed this institutional ethnographic study at 13 healthcare facilities in Gauteng province. We developed an observation guide to explore the intersection of culture and environment and its influence on healthcare provision to patients, especially migrants. Following ethics approval, we observed the facilities for 234 person-days. We used thematic analysis to analyse the data.

**Results:**

Busy, frantic or nervous spaces, and contestations between patients and health workers, and among health workers formed part of the social and cultural environment of healthcare provision. The presence of migrant patients during busy periods served as a detonator for rude or discriminatory remarks, exacerbated by staff shortages and language barriers. Simultaneously, migrants exercised their agency by rebutting or confronting rude health workers. We also observed encouraging examples of kindness, caring and professionalism of health workers.

**Conclusion:**

The study has implications for achieving a migrant-sensitive health system in South Africa.

## PAPER CONTEXT

- **Main findings:** The ethnographic observations at public health facilities demonstrated the duality of social exclusionary behaviours of some healthcare providers, while many others provided healthcare with professionalism, kindness, and compassion.
- **Added knowledge:** The study generated new knowledge on the influence of the environment on service delivery within the context of health systems responsiveness, and social exclusion.
- **Global health impact for policy and action:** The findings contribute to the global discourse on the provision of migrant-sensitive universal health coverage.

## Background

Central to the global discourse on universal health coverage (UHC) that combines the principles of equity, need, and financial risk protection, is the notion of a migrant-sensitive health system [1]. The latter implies the explicit and systematic incorporation of migrant needs into health policy, financing, and service delivery [2]. Notwithstanding encouraging health programmes [2], migrants continue to have limited healthcare access, they face multiple barriers, and/or are excluded from national health systems regardless of country context or income levels [3–9].

Within the discourse on UHC, health systems responsiveness (HSR), a measure of the performance of the health system and its ability to meet the expectations of individuals when using health services [10], has assumed increasing importance. HSR focuses on eight domains of dignity, autonomy to participate in health-related decisions, confidentiality, prompt attention, adequate quality of care or basic amenities, communication, access to social support networks, and choice of healthcare worker [11]. Social scientists have underscored the potential and benefits of ethnographic studies in enhancing our knowledge and understanding of HSR and the intricacies and dynamics of healthcare access for migrants [12,13]. Consequently, some scholars have focused on local institutions, their responsiveness to the needs of migrants, the environment of care provision, and/or the behaviours of health policy actors [14–16].

South Africa has a rights-based Constitution that prohibits discrimination [17]. However, evidence points to a failure and/or inability of the health system to recognise and address the health needs of migrants, especially those from other African countries [18,19]. This is partly due to the country’s complex history of apartheid, discrimination and disenfranchisement of the majority of South Africans, and a cheap migrant labour system [20]. This legacy of discrimination and human rights violations of black international migrants fundamentally shaped immigration policies and societal attitudes in the post-apartheid period [21]. Nonetheless, South Africa is the top destination for migrants from other African countries, with an estimated 2.4 million living in the country in 2022 [22].

Although several studies in South Africa have highlighted migrants’ experiences of discrimination, social exclusion and medical xenophobia in the public health sector [23–25], Vanyoro has illustrated the diverse and positive experiences of migrants in the South African public health system [26]. However, there is a dearth of ethnographic studies on HSR and migrants in public health facilities. Consequently, the aim of this study was to explore the environment of healthcare provision and the everyday practices and behaviours of health workers and patients at a selection of healthcare facilities in the Gauteng province of South Africa, with a specific focus on international migrants. This is because healthcare for international migrants cannot be separated from the overall context of the public service provision in South Africa. In this study, a migrant is defined as ‘any person who is moving or has moved across an international border or within a country away from the habitual place of residence, regardless of the person’s legal status; whether the movement is voluntary or involuntary; and/or the causes of the movement’ [27]. Environment refers to the physical, social and cultural aspects of healthcare provision [28,29].

## Methods

### Conceptual framework

In this study, we used the HSR conceptual framework of Mirzoev and Kane, and the theory of social exclusion [30] to observe the intersection between the everyday behaviours and practices of health workers and patients and the environment of healthcare provision in public health facilities. Mirzoev and Kane’s framework positions people’s experiences of dignified treatment, autonomy, confidentiality, prompt attention, access to networks, quality of amenities, choice of provider, and trust at its core [31]. The framework recognises that health policy actors, health service delivery processes, and organisational arrangements shape the experiences of health service users or patients [31].

This HSR conceptual framework resonates with the definition of social exclusion as a dynamic, relational concept in health system, the centrality of people and power relationships, and the intersections of these with cultural, economic, political and social dimensions [30].

The combined conceptual framework informed this ethnographic study at 13 health facilities in Gauteng Province [30,31]. We observed five of the eight core patient experiences of dignified treatment, autonomy, confidentiality, prompt attention, and quality of amenities, as it was neither practical nor possible to observe access to networks, choice of provider and trust [31]. We also observed the healthcare delivery processes, behaviours, communication, and interactions between and among patients and health workers.

### Study setting

Gauteng was selected because of its strategic importance in South Africa, with an estimated inflow of international migrants of around 1.4 million between 2021–2026 [32], the ability to recruit international migrants for the study, geographic proximity, established relationships with healthcare authorities, and budget considerations.

In 2018, there were 354 healthcare facilities in Gauteng categorised as: primary healthcare (PHC) clinic (*n* = 290), community health centre (*n* = 30), district hospital (*n* = 11), regional hospital (*n* = 9), central hospital (*n* = 4), regional tertiary hospital (*n* = 4) and specialised mother and child hospital (*n* = 1). We used stratified, random sampling to select two facilities from these categories, with the exception of the mother and child hospital (*n* = 1). Hence, 13 health facilities were selected, described in detail in two previous publications [15,25].

### Study design

This was an institutional ethnographic study, an approach to social research that uses everyday experiences as entry points, and aims to explore how peoples’ daily activities are influenced by different institutions [33], in this study the 13 selected public healthcare facilities. In concert with traditional ethnography [33], we used observations and immersion for 234 person-days, complemented by detailed field notes and informal conversations to explore the environment of healthcare provision and the everyday practices and behaviours of health workers and patients.

### Ethnographic observation guide

Drawing on the combined conceptual framework [30,31], we developed an ethnographic observation guide to answer the following research questions. Firstly, what is the environment (physical, social and cultural) of healthcare provision at each clinic or hospital? Secondly, what are the behaviours and practices of health workers and patients (especially international migrant patients) i.e. the culture, in each health facility? Thirdly, how does the culture and environment intersect, and how does it affect healthcare provision to patients, specifically, migrant patients?

The physical environment encompassed observation of the size of the waiting room, sufficient seating for patients, the availability of drinking water, and the cleanliness of the health facilities. The social and cultural environment captured the behaviours of patients and health workers; and the atmosphere of the environment, using concepts of tension and busyness drawn from the piloting for other components of the larger doctoral study [34]. In this study, health workers refer to any staff member who contributes to healthcare provision, whether directly (such as nurses and doctors), or indirectly (such as clerks, cleaners, and security personnel). We use the term healthcare provider when the person is a trained health professional such as a nurse or doctor. We also captured public conversations or comments made to the researchers. Table 1 shows the observation guide for the study, and its linkages to the conceptual frameworks.

**Table 1.** Observation guide.

Conceptual framework	Research question	Observation guide
Health Systems Responsiveness (HSR) – quality of amenities	What is the physical environment of health care provision at each clinic or hospital?	• What is the size of the waiting room? • Is there seating for patients, e.g., chairs? • Are there enough chairs or sufficient seating? • Is there drinking water available? • Is the waiting room and/or health facility clean?
HSR – dignified treatment, processes, actors	What is the social and cultural environment of care provision? What are the interactions between and among health workers and patients?	• Who is sitting (men, women, South Africans, non-South Africans, old, young, pregnant?)? • Who is standing? • Are people treated with respect? • Are there obvious exclusionary practices e.g. labelling, name-calling?
HSR – prompt attention, processes, actors		• Are sick people attended to? • Which categories of health workers are visible in the facility? What are they doing or saying?
HSR–autonomy, confidentiality, processes, power relations, social exclusion		• Are questions asked in a respectful manner? • Are people treated in privacy? • What is the atmosphere in the waiting room or in the health facility (calm, tense, busy, frantic)? • What are the public conversations or comments in the waiting areas? • Are there obvious exclusionary practices, e.g. labelling, name-calling?

### Data collection

The data was collected between April 2018 and December 2018, with a total observation time of 234 person-days. The research team focused the observations on entry points, waiting areas, and the exit points of the health facilities.

The public spaces did not breach or interfere with health provider-patient confidentiality and allowed the research team to blend into the environment. On all fieldwork days, the Principal Researcher (PR) and fieldwork team each completed their own observation guides (see Table 1). We observed the physical environment, the social and cultural environment of care provision, especially the interaction between health workers and international migrants. We identified international migrants based on the language spoken, their accents and/or traditional attire or dress code. We endeavoured to be unobtrusive and listened to public conversations or comments in the waiting areas. We observed whether there were any labelling and/or name-calling of international migrants. Additionally, each fieldworker took detailed notes in field diaries.

### Analysis

All facility identifiers were removed, and each allocated a unique number. The PR consolidated all observation guides and fieldwork notes into a spreadsheet. The observation guides and field-notes were analysed using thematic analysis [35]. The authors (JW & LR) read the consolidated information without any preconceived notions or classification. We then examined the information in light of the study’s research questions.

Four experienced researchers participated in the development of codes by independently reading the consolidated field notes for all 13 health facilities, thereby ensuring reliability and establishing inter-coder agreement. Once the initial analysis was completed, the team met to discuss the codes that were independently generated. Following this initial discussion, the team generated sub-themes and themes and reached agreement on these (Table 2). The PR then extracted the key messages within those broader themes.

**Table 2.** Themes from ethnographic observations at health facilities.

Theme	Sub-themes
Health care context, environment and spaces	• Variations in cleanliness. • Littering in health facilities. • Variations in availability of basic amenities (e.g. water, tissue paper). • Insufficient seating. • Overcrowding. • Insufficient number of consultation rooms. • Lack of privacy. • Tense or nervous atmosphere.
Patients, patience and agency	• Large number of patients. • Diversity in number of migrants at different facilities. • Expressed dissatisfaction or anger because of delays in, or lack of care provision. • Leaving facilities before consultation. • Refusal of urgent medical treatment. • Confronting rude or unacceptable health worker behaviour.
Health workers, ethics and street level bureaucrats	• Caring and empathy. • Staff shortages. • Perceived lack of urgency for emergencies. • Language barriers. • Contestations among health workers. • Disrespectful and unprofessional behaviour. • Expressed stereotypes or xenophobic views about migrants.
Variations and ambivalence in health care provision	• Variations in waiting times. • Structured and well-organised systems of triage or waiting. • Disrespectful or patronising verbal communication. • Denial of care because of lack of documentation. • Deservingness of migrants to utilise public health facilities.

Credibility and trustworthiness were established through inter-coder agreement and the independent coding of a sample of observations. The use of trained observers and a standard observation guide enhanced the consistency and reliability of our observations while minimising bias [36]. We also used conceptual, methodological and data triangulation by comparing the results obtained from the ethnographic observations with the results of the policy analysis [37] and the two surveys [25,34].

### Ethical considerations, researcher conduct and positionality

The study was guided by the principles contained in the Singapore Statement on Research Integrity [38], and all ethical procedures were adhered to. The Human Ethics Research Committee (HREC) Medical of the University of the Witwatersrand provided ethical approval for the study. Both the Gauteng Department of Health and the City of Johannesburg municipality granted permission to access health facilities for fieldwork.

The ethical principles included the voluntary participation of all participants and ensuring confidentiality and anonymity of all participants and study information. We asked health workers and patients to indicate any objections to our presence and observations, to which there were none. We assigned an identifier to each healthcare facility to ensure anonymity and confidentiality. The health workers or patients referred to in the observation guides or field notes through public conversations remain anonymous. The data is stored on a password-protected computer.

We undertook non-participant observations, hence we did not directly engage with patients and health workers. Non-participant observations may provide a more objective view of the social dynamics at play [39], within the environment of healthcare provision. Although identifying international migrants by their language, accents or dress code has the potential for bias, the option of asking people for their country of origin could have elicited potential negative consequences for the individuals concerned. We were transparent about the role of the research team and emphasised that the data would be reported anonymously and there would be no benefits to, or risks of, taking part in the study.

Both authors are women of colour, committed to social justice and human rights, in relatively privileged positions of employment at a prestigious university. Hence, an important part of conducting the research study, was to ensure reflexivity [40], and how this might influence the research study. Data analysis was an iterative process, and combined with reflexivity, to ensure that any potential biases did not influence the findings.

## Results

Four, overlapping themes emerged from the analysis of our data: (1) context, environment, and public spaces; (2) patients, patience, and agency and behaviour; (3) health workers, ethics and street level bureaucrats; and (4) variations and ambivalence in healthcare provision (Table 2).

### Theme 1: context, environment, and public spaces

This theme comprises the physical environment and the public spaces of healthcare provision and interactions among health workers and patients. We observed that the 13 health facilities functioned as vibrant social spaces, with vendors selling various items to a captive audience of patients as they waited for their healthcare consultations. Health service users or patients connected with each other through informal conversations.

Our observations revealed variations in the cleanliness and the availability of basic amenities. Drinking water was mostly available. Each observation day commenced with a clean and relatively litter-free environment. However, the cleanliness of the waiting areas and public bathrooms changed throughout the day, influenced by the [in] activities of the cleaners and littering by patients. We observed appeals from healthcare providers to patients in waiting areas to use the trash bins for their litter. In some instances, the behaviour of health workers also influenced the cleanliness of facilities. At HF8, we observed that used intravenous tubing was discarded into the bin of the public bathroom, instead of the medical waste container. At five of the health facilities (HF3, HF5, HF8, HF10, HF11), we observed a lack of tissue paper in the public bathrooms, as the day progressed.

The age of and infrastructure at the 13 facilities determined the adequacy of waiting areas and seating arrangements. At the PHC clinics, there was an insufficient number of consultation rooms. At HF1 and HF2, the healthcare providers were obliged to share consultation rooms on busy days. This resulted in a lack of privacy and breach of confidentiality during patient examination. At HF1, we observed a healthcare provider taking a blood pressure of a female patient in the waiting area in full view of others present. As the woman had to undress partially to fit the cuff of the sphygmomanometer, her right to privacy was violated. Similarly, the scale for weighing patients was placed at the door leading into the main reception area, thus compromising confidentiality.

On busy days, which varied depending on the type of facility, there were large numbers of patients seeking care, contributing to overcrowding and noise in the waiting areas. Such overcrowding in turn influenced the atmosphere in the health facility. We observed both nervous patients, often unsure of their turn for health provider consultation, and tense or irritable healthcare providers, when faced with an overcrowded waiting room. The atmosphere was also influenced by the location of the outpatient department in the health facility. Those outpatient departments close to emergency (casualty) departments in hospitals appeared chaotic, especially when seriously ill or injured individuals arrived. The presence of migrant patients during the very busy periods served as a detonator for rude or discriminatory remarks, such as observed at HF1, HF3, HF4 and HF9. In contrast, the spaces in health facilities appeared quiet and calm when there were fewer patients.

### Theme 2: patients, patience, and agency and behaviour

We observed that the selected health facilities were busy, with large numbers of individuals seeking healthcare. Although many health facility managers reported that ‘foreigners’ constituted the majority of patients, our observations revealed variations across the 13 facilities, and did not support the notion that migrants comprised the majority of patients. Instead, we found a mixed patient population with varying numbers of South Africans and international migrants in the selected healthcare facilities. In some facilities (HF1, HF3, HF4, and HF9), the research team recruited a large number of migrant patients on the three randomly selected fieldwork days, whereas a small number of migrant patients were recruited at the remainder.

During our fieldwork, many patients expressed dissatisfaction or anger because of delays in, or lack of care provision. At HF1, a female migrant, complained that she had been at the clinic for several hours. She decided to leave to go to work, without consulting with a healthcare provider, and indicated that she would try to return to the clinic the following week. At the same HF1, another female migrant left with her sick child before the healthcare consultation because of lack of attention by healthcare providers.

At HF5, we witnessed a patient’s refusal of urgent medical treatment, despite the explanation and pleas from healthcare providers for referral to a central hospital. At some facilities, we observed that patients exercised agency by confronting rude or unacceptable behaviour. At HF7, a female migrant patient rebutted a healthcare provider who told her that she was not sick because she looked beautiful. At HF10, another female migrant patient confronted a nurse in the triage section who scolded her for coming to the hospital dressed in a nightgown.

### Theme 3: health workers, ethics, and street level bureaucrats

Our observations revealed that the interactions between healthcare providers and patients ranged from professionalism, compassion, and empathy to disrespectful and unprofessional behaviour. In some instances, health workers made outright discriminatory comments towards migrant patients.

At HF4, the kind, professional and compassionate behaviour of a nurse was striking. She regularly monitored the parents and their children in the waiting area, encouraged parents to speak up if their children needed urgent attention. In HF2, nurses and other staff members performed their respective duties, and the interaction was cordial. In many of the health facilities, we observed that cleaners swept or polished floors to ensure a clean and hygienic environment.

The behaviour of healthcare providers, and their interactions with patients were influenced by staff shortages and resource availability. Language barriers challenged both healthcare providers and patients in different ways. At HF9, we observed a migrant patient struggling to explain her symptoms, resulting in the nurses getting irritated and shouting at her.

However, even when the same language is spoken, barriers exist between the use of technical language or terms, and the comprehension of patients. At HF7, nurses tried to teach female patients about condom use, but the nurses got impatient and started shouting when the patients did not engage with them during the demonstration.

In some facilities, the tensions, insufficient teamwork, or lack of collaboration boiled over, exacerbated by the hierarchical nature of the healthcare system. At HF1, a clerk informed the nurses in the tearoom about an estimated 40 patients in the waiting room. He threatened to escalate the lack of attention to patients to the PHC clinic facility manager. One of the nurses reprimanded the clerk and asked him, ‘*Who do you think you are*’.

Our observations demonstrated some healthcare providers’ disrespect, and prejudicial views about migrant patients. At HF1, we observed the interaction between the facility manager (a professional nurse) and a migrant patient in the main reception area. The facility manager turned to a woman with a child on her lap and addressed her in isiZulu [one of South Africa’s official, indigenous languages]. When the woman replied in English, the health provider told her ‘I’m going to deport you’ and laughed. At both HF2 and HF11, some healthcare providers told the researchers that migrant patients demanded a three months’ supply of medicines with the intention of selling the medicines or sending it to family and friends living in their home countries. They also alleged that migrants obtained South African identity documents fraudulently in order to access health services.

### Theme 4: variations and ambivalence in healthcare provision

Waiting times in the healthcare facilities varied a great deal, influenced by the number of health workers at a facility relative to patient numbers and the internal hospital systems for managing queues. At HF5, we observed an excellent example of structured and automated patient queueing systems. There were clearly demarcated areas for the provision of different healthcare services. Patients were able to hear when their number was called and to see the designated consultation room in the hospital. In contrast, at some of the other clinics and hospitals (HF3, HF8, HF9, HF10, HF11), we noticed that the poor organisation of patient queues and the lack of information about the different queues. This in turn led to long waiting times. When patients were in the wrong queue, this further increased their waiting time.

We observed that verbal communication could provide useful information and be patronising and potentially disrespectful at the same time. At HF1, a nurse reminded parents about the specific clinic days for vaccinations. She addressed the patients in the waiting area and asked them why they were throwing litter on the floor when there was a dustbin for trash. Lastly, the nurse talked to the patients about the nutrition and feeding of their children. She stated publicly that:

Young ladies are lazy to make soft porridge … if you cannot cook for the baby don’t make the baby. I love babies, when I fight with you, I’m fighting for the baby.

At the same facility, the general assistant responsible for maintenance shouted at a migrant woman because her son was playing in the parking space. An argument ensued, and the general assistant told the woman that she should ‘*go home*’. At HF9, a healthcare provider remarked to the migrant pregnant women in the waiting room that ‘*all of you come here* [to South Africa] *when you have to give birth*’.

At HF13, we observed that a migrant patient was turned away because of lack of documentation. The attending nurse explained that emergency cases are not required to produce documentation, but that documentation is required when the individuals returned for a follow-up appointment.

We observed another example of a healthcare provider’s judgement about the deservingness of migrants to utilise public health services. A migrant woman with a sick child was kept waiting for 30 minutes for the consultation at HF4, although there was no one in the emergency section of the facility at 5 pm in the afternoon. The health worker at the reception desk asked the woman why she waited the whole day to come to the facility. The migrant patient replied that she was working and could only bring her child for a consultation at that time. Eventually, the woman was called into the consultation room when the healthcare provider considered it appropriate to see her.

## Discussion

In this institutional ethnographic study, we combined the lens of HSR and social exclusion to explore the intersection of the environment in which care is delivered for migrants and the everyday practices and behaviours of health workers and patients.

We found that ageing and unfit-for-purpose health facility infrastructure contributed to sub-optimal patient experiences of dignified treatment and confidentiality. The violation of patients’ privacy and confidentiality was stark at the PHC clinics, where nurses shared consultation rooms and where there was no space for patient screening and/or weight measurement. Although a different context, a systematic review of migrant women’s experiences of pregnancy, childbirth and maternity care in European countries found that women associated quality care with health facilities that promoted privacy [41]. In South Africa, the infrastructural constraints at PHC level have led to substantial government investment in the Ideal Clinic Realisation and Maintenance Programme to improve the quality of healthcare [42]. Although the building or addition of new rooms to a clinic is outside the remit of an individual PHC facility manager, a core responsibility is to ensure the provision of holistic and comprehensive care to patients [43]. Hence, it should be possible to find a way to re-organise services to ensure greater privacy and confidentiality, especially during busy periods. A possible solution could be to use a mobile screen for greater privacy within the PHC clinic. Furthermore, an appointment system to improve patient flow and reduce crowding in waiting areas should also be considered. The PHC facility manager could also enlist the help of the clinic committee to raise funds for a temporary structure that could be used for the screening of patients.

Our study found that HSR was influenced by variations in facility cleanliness and the availability of basic amenities that intersected with the negative behaviours and actions of patients and health workers. Although cleanliness appears straightforward, patients who littered, lax cleaners, or inappropriate disposal of medical waste made it difficult to maintain health facility hygiene, a core element of HSR. A 2017 hSR study in Ethiopian health facilities found that the domain of basic amenities obtained the lowest scores and did not meet patient expectations [44]. In South Africa, cleanliness of health facilities remains one of the priority areas for inspection by the Office of Health Standards Compliance [45]. The 2018/19 inspection report confirms our ethnographic observations regarding cleanliness, as hospitals and CHCs obtained average cleanliness scores of 68% and 65%, respectively, while clinics scored only 48% [45]. Our ethnographic study suggests that cleanliness and improvements of basic amenities require a comprehensive approach that involves patients and all health workers. At health facility level, there should be improved supervision of the cleaning staff, and sharing of best practices as some of these facilities remained spotless even on busy days. Patients and the community at large should also be educated on the importance or cleanliness, linked to broader environmental awareness campaigns.

We heard a common narrative that international migrants both overwhelm and exploit the South African public healthcare system, exacerbating resource constraints and the difficulties of providing quality healthcare. This narrative was repeated by the public comments from health workers at some of the health facilities, who also expressed xenophobic stereotypes about migrants. Similarly, reports from non-governmental organisations and the media highlight the xenophobic stereotypes, with many politicians and communities blaming international migrants for a host of social ills, including criminal activities, poor service delivery and exploitation of the public healthcare system in South Africa [46]. Other scholars in South America and the United Kingdom have also highlighted this narrative of the exploitative and fraudulent migrant [47,48].

However, during the 69 days that we spent at the 13 facilities, we did not observe the ‘busloads of migrants’ that arrived for healthcare. Instead, we found variations in the number of international migrants who utilised the 13 facilities. At some facilities, migrant patients constituted a large proportion of people utilising health services, while at others there were very few migrants. The variation in numbers could be explained by the geographic location of health facilities and the surrounding neighbourhoods where migrants from the same country of origin choose to settle. Although we could have missed the phenomenon of the ‘busloads of migrants’ by chance, the narrative of the healthcare system overwhelmed or exploited by migrants could be explained by the ongoing xenophobic political rhetoric [46]. Importantly, several scholars have highlighted the complex factors that explain xenophobia in South Africa. These include the myths and stereotypes about foreigners, caused by the disjuncture between South Africa’s progressive laws and implementation, the apartheid legacy of racism directed against African migrants, feelings of superiority, a fear of losing social status and identity, and the perceived threat of foreigners competing with locals for scarce resources and public services [49]. Nonetheless, it is the responsibility of health policy-makers to ensure that all actors, including those in the health system, uphold the human rights of migrants in line with the Constitution [17]. The various regulatory bodies that govern health professions stipulate their moral and ethical duties and obligations [50], including the principle of non-discrimination. While it is crucial that regulatory bodies remind healthcare workers of their ethical obligations and to sanction those who fail to uphold their ethical and professional obligations, the systemic challenges faced by health workers should also be addressed. There must be a concerted effort to provide the resources, training and support necessary for healthcare workers to provide responsive and compassionate services to all patients.

In this study, busy, frantic, or nervous spaces, and contestations between patients and health workers, and among health workers formed part of the social and cultural environment of healthcare provision. At HF1, we observed the tension between nurses and the clinic clerk who threatened to report the nurses to their managers, because of the large number of patients waiting to be seen. We could not find other South African studies that have explored the relationship between nurses and clerical staff. A 2005 study at a US hospital found that a tension exists between ‘the ideal democratic team structure and the reality of a healthcare hierarchy created by educational differences and traditionally superior – subordinate positions’ [51,p.103].

The observed interaction between the facility manager and migrant patient at HF1 highlights several important issues. Firstly, the interaction illustrates how language as an identifier [of international migrants], and the power of a health worker to mediate healthcare access, combined to stigmatise migrant patients. Secondly, it highlights migrant patients’ vulnerability when utilising public healthcare services in South Africa, particularly those differences in language and the pathway to vulnerability within the healthcare context. This interaction between language and vulnerability was also illustrated in a Swedish study among immigrant women and their healthcare experiences [41].

Thirdly, it underscores the negative power of the street-level bureaucrat, in this case the healthcare provider [52]. Lipsky has argued that frontline staff exercise their discretion about ‘which patients they will help’. This discretion intersects with negative power and discriminatory attitudes about migrant patients, thus influencing HSR negatively. Conversely, a Norwegian study showed the positive role of the street-level bureaucrat who acted as an agent for accessibility to the Norwegian healthcare system and support for immigrant women utilising maternal healthcare services [53]. More recently, scholars have emphasised the complex moral judgements made by healthcare providers, influenced by their assumptions about those individuals who deserve access to care [4,54]. In this study, the notion of deservingness was illustrated by the healthcare providers’ judgements on whether the sick children of the migrant women at HF1 and HF4 were worthy to receive care. Furthermore, using the social exclusion lens, the interaction also illustrates language as an identifier and as a mechanism for social exclusion [30] during the healthcare encounter. Hence, the intersection of street-level bureaucracy [52], deservingness [54] and social exclusion [30] produced poor HSR for these two migrant women, and their children.

In this study, migrants exercised their agency in different ways. At several of the health facilities, migrant patients rebutted or confronted health workers, despite the potential risks of being refused care or facing verbal abuse. In some instances, they left after long waiting times, with the expressed intention to return at a later stage. However, leaving the facility is not an option for sick patients, who might need immediate medical attention. A complementary survey among migrant patients at these 13 facilities found complex and nuanced health service experiences, with their perceptions of HSR closely linked to their satisfaction with health workers [25].

Our observations of disrespectful encounters between health workers and migrant patients and their expressed stereotypes or xenophobic views resonate with the findings of other South African studies that highlighted migrants’ experiences of medical xenophobia and discrimination [23,55]. In a complementary survey among healthcare providers at the 13 health facilities, 19.2% of healthcare providers reported that they had witnessed discrimination against migrants, while 20.0% reported differential treatment of migrant patients [15].

Paradoxically, we observed examples of health worker kindness, caring and professionalism in the studied health facilities. This was also found in the survey among migrant patients [25], highlighting the multiple narratives of healthcare provision for migrants in South Africa. These examples provide hope and a counter-narrative to the challenge of exclusionary healthcare practices in South Africa. Similarly, Vanyoro, found that health workers in another South African Province endeavoured to provide migrant-friendly healthcare through language adaptations [26]. Hence, an inclusive and human rights approach to healthcare provision for migrants is likely to result in benefits for all health service users, despite the resource constraints in South Africa’s public health sector [18].

Our ethnographic observations were limited to public spaces and outpatients and emergency departments of the selected healthcare facilities. We did not observe the environment and interactions within consultation rooms or the wards, where different interactions might have been observed. Another limitation is that the study was conducted in the Gauteng province of South Africa. Hence, the findings cannot be generalised to the healthcare facilities in other South African provinces. However, Gauteng is host to 47.5% of international migrants in South Africa [56]. Hence, this study provides important insights into the environment in which care is delivered and the daily practices and behaviours of health workers and patients, including migrants.

Conducting institutional ethnographic research among vulnerable groups raises ethical dilemmas. One of the dilemmas is whether the research team could or should have intervened with the incidents of discrimination. Or would it have caused more harm? The answer is that we do not know. Another dilemma is the risk of perceived interference in healthcare operations, with consequences for other researchers.

Nonetheless, ethnographic observation adds value when done as part of an overall mixed methods approach to explore the experiences of migrants in the health system of host countries [57]. The prolonged immersion and engagement allowed us to familiarise ourselves with the physical, social and cultural environment of healthcare provision, and its intersections with patients and health workers [57]. At the same time, such engagement is time-consuming, necessitating a well-planned schedule for fieldwork, and careful considerations of logistics and resource requirements.

A major strength of our study is combining the conceptual frameworks on HSR and social exclusion to explore the environment of healthcare provision for migrant patients in the public health facilities of Gauteng.

## Conclusions

Using the concepts of HSR and social exclusion, our study demonstrates the influence of the physical, social, and cultural environment on healthcare provision for patients, particularly migrant patients, at 13 public healthcare facilities in the Gauteng Province of South Africa. Our study draws attention to the multiple narratives in the provision of healthcare to all patients, including migrants. The professional conduct of health workers who uphold their duties is encouraging, while the disrespectful encounters between health workers and migrant patients require attention and should be sanctioned. The social and cultural environment, through busy, frantic, or nervous spaces, and the contestations between patients and health workers, and among health workers further complicate the provision of healthcare.

The pandemic has underscored the intersection of pre-existing health inequities, problematic immigration policies and denial of social services for international migrants living in South Africa [58]. Our findings highlight the persistent challenges in achieving people-centred, responsive healthcare, a core element of universal health coverage, and the tensions and the practical difficulties of delivering quality care in resource-constrained settings. Simultaneously, the study findings highlight the potential of achieving a migrant-sensitive health system because of the observed kindness, care, and professionalism of many health workers.
